# Association synchrone de deux tumeurs pulmonaires primitives: à propos d’un cas

**DOI:** 10.11604/pamj.2023.45.174.31477

**Published:** 2023-08-21

**Authors:** Ahmed Id M´barek, Hatim Kouismi

**Affiliations:** 1 Service de Pneumologie, Université Mohamed Premier d´Oujda, Faculté de Médecine et de Pharmacie d´Oujda, Centre Hospitalier Universitaire Mohamed VI, Oujda, Maroc

**Keywords:** Tumeurs pulmonaires synchrones, adénocarcinome, carcinome épidermoïde, cas clinique, Synchronous lung tumors, adenocarcinoma, epidermoid carcinoma, case report

## Abstract

Les tumeurs pulmonaires multiples primitives synchrones représentent une entité relativement rare avec une incidence en augmentation croissante ces dernières années grâce au développement des moyens de l´imagerie thoracique et des techniques de l´immunohistochimie. La deuxième lésion est considérée dans la plupart des cas comme une localisation secondaire ce qui explique en partie la baisse de l´incidence de cette entité. Nous rapportons l´observation d´un patient âgé de 74 ans présentant deux tumeurs pulmonaires primitives synchrones, un adénocarcinome et un carcinome épidermoïde. A travers cette observation clinique nous soulignons la difficulté du diagnostic des tumeurs synchrones et sur l´intérêt majeur des nouvelles modalités d´imagerie et de l´immunohistochimie pour la prise en charge optimale de ces tumeurs.

## Abstract (en)

Synchronous multiple primary lung tumors are a relatively rare entity with an increasing incidence in recent years due to the development of thoracic imaging and immunohistochemical techniques. The second lesion is considered in most cases as a secondary location, which partly explains the decrease in the incidence of this entity. We report the observation of a 74-year-old patient with two synchronous primary lung tumors, an adenocarcinoma and an epidermoid carcinoma. Through this clinical observation, we highlight the difficulty of diagnosing synchronous tumors and the major interest of new imaging modalities and immunohistochemical techniques for the optimal management of these tumors.

## Introduction

Les tumeurs bronchiques primitives multiples synchrones sont définies comme des tumeurs distinctes d´origine bronchopulmonaires, découvertes au même moment et chacune de ces tumeurs présente une histologie différente [1]. Ces tumeurs synchrones représentent une entité très rare avec une incidence qui varie entre 0,2 et 8% [2]. Nous rapportons le cas d´un patient avec deux tumeurs bronchiques primitives synchrones.

## Patient et observation

**Informations du patient:** c´est un homme âgé de 74 ans sans antécédent pathologique notable, tabagique chronique à raison de 40 paquets/année, qui présentait des douleurs latéro-thoraciques droites évoluant depuis 5 mois aggravé par l´apparition d´une hémoptysie de faible abondance, le tout évoluant dans un contexte d´amaigrissement non chiffré.

**Résultats cliniques:** l´examen clinique trouvait un patient stable sur le plan hémodynamique et respiratoire avec un indice de performance de l´Organisation mondiale de la Santé (OMS) chiffré à 1, le reste de l´examen physique était sans particularité.

**Figure 1:**
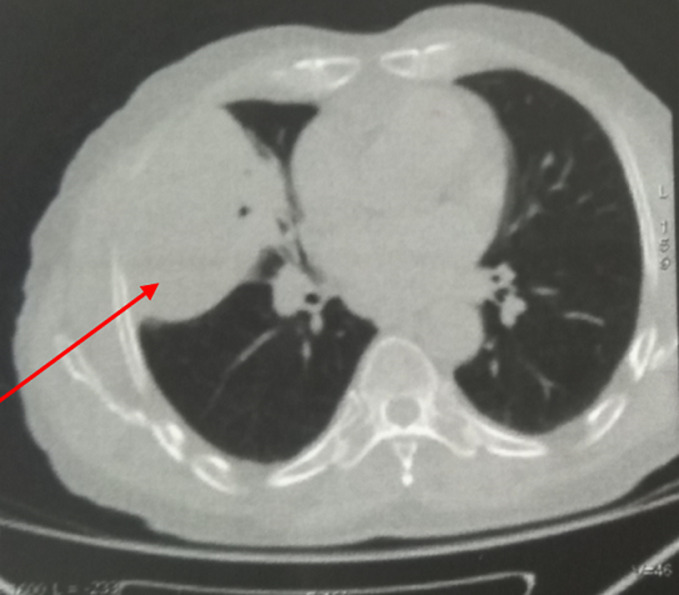
coupe axiale du scanner thoracique montrant une masse pulmonaire droite

**Figure 2:**
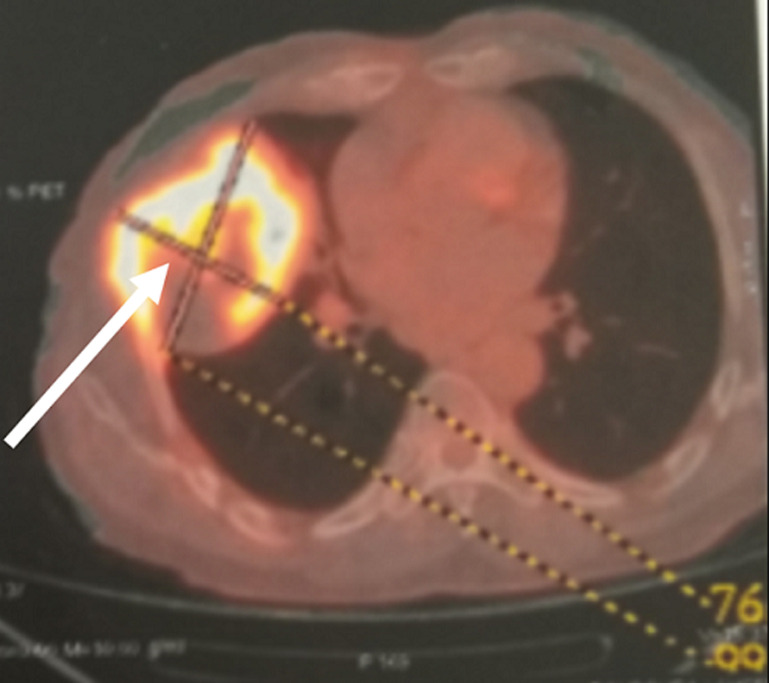
tomographie par émission de positrons montrant une masse pulmonaire droite hypermetabolique

**Figure 3:**
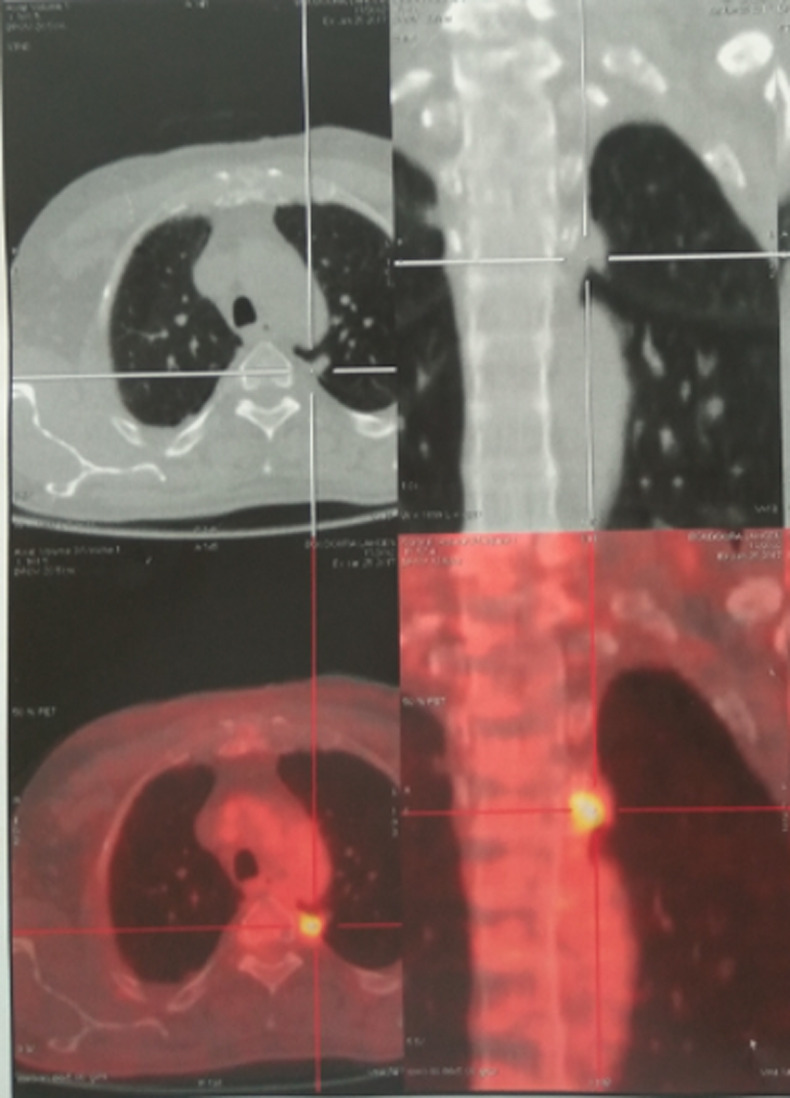
tomographie par émission de positrons montrant un nodule hypermétabolique au niveau du segment postérieur du lobe supérieur gauche

**Démarche diagnostique:** la radiographie thoracique montrait une opacité hétérogène du champs pulmonaire droit. Le scanner thoracique objectivait un processus parenchymateux pulmonaire mesurant 100x76 mm à cheval entre le lobe moyen le lobe inférieur du poumon droit (Figure 1). La bronchoscopie souple n´a pas montré de lésion endobronchique en dehors d´une inflammation diffuse. Une biopsie sous guidage échographique de la masse a été faite et dont l´étude histologique était en faveur d´un carcinome épidermoïde bien différencié, kératinisant et infiltrant. Le bilan d´extension fait n´a pas révélé de localisation secondaire. La tomographie par émission de positrons au 18 Fludeoxyglucose (FDG) a objectivé en plus de la masse active décrite au scanner thoracique (Figure 2) un nodule actif suspect du poumon controlatéral au niveau du segment postérieur du lobe supérieur gauche hypermétabolique (SUVmax: 4.7) mesurant 10x9 mm (Figure 3), sans atteintes ganglionnaires ou autre foyer suspect. Une thoracotomie avec curage ganglionnaire et résection du nodule suspect a été faite et dont l´étude immuno-histologique du nodule était en faveur d´un adénocarcinome d´origine bronchopulmonaire.

**Intervention thérapeutique et suivi:** le patient a bénéficié par la suite d´une bi-lobectomie droite. L´évolution était rapidement défavorable avec décès du patient au huitième jours post opératoire.

**Consentement du patient:** le patient a donné son consentement pour la rédaction et la publication de son cas.

## Discussion

Les tumeurs pulmonaires multiples peuvent être synchrones (diagnostiqué simultanément) ou métachrone (diagnostiqué à des moments différents) [3]. La première classification et description des critères diagnostiques des tumeurs bronchiques primitives multiples synchrones (TBPMS) a été établie en 1975 par Martini *et al*. [1]. Cette classification définie les TBPMS comme étant deux ou plusieurs tumeurs distinctes qui se développent indépendamment au cours de la même période et présentant une histologie différente de celle de la tumeur primaire. Dans la littérature l´incidence d´un double cancer primitif du poumon synchrone est estimée entre 0,2 et 20% [4]. L´incidence exacte est difficile à déterminer vu que la deuxième lésion est souvent considérée comme une métastase de la tumeur primitive et ceci est expliquée essentiellement par la limite d´utilisation ou la non-disponibilité des moyens et techniques de diagnostic notamment la tomographie par émission de positrons [5]. Parfois la distinction entre les tumeurs bronchiques primitives et les lésions secondaires métastatiques est difficile et constitue un défi majeur pour les anatomopathologistes mais cette distinction reste essentielle vu que la différence en matière de prise en charge diagnostique, thérapeutique et pronostique [6]. La découverte de multiple cancer pulmonaires synchrones impose une prise en charge de chaque tumeur de façon séparée et distincte en matière de stadification et du traitement [7]. Notre patient présentait deux lésions primitives: un carcinome épidermoïde bien différencié au niveau du poumon droit (lobe moyen et inférieur) et un adénocarcinome au niveau du lobe supérieur gauche et la tomographie par émission de positons était très utile pour la stadification et la prise en charge thérapeutique.

## Conclusion

L´incidence des tumeurs bronchiques synchrones est en augmentation vu le développement des techniques de l´imagerie et des autres modalités diagnostiques. Nous insistons à travers cette observation sur l´intérêt d´essayer d´avoir une preuve histologique surtout en cas de métastase pulmonaire unique et également sur la nécessité de la biologie moléculaire afin de comprendre les mécanismes physiopathologiques de cette association synchrone.
